# Energy Coupling in Cation-Pumping Pyrophosphatase—Back to Mitchell

**DOI:** 10.3389/fpls.2020.00107

**Published:** 2020-02-14

**Authors:** Alexander A. Baykov

**Affiliations:** Belozersky Institute of Physico-Chemical Biology, Lomonosov Moscow State University, Moscow, Russia

**Keywords:** membrane pyrophosphatase, H^+^ pumping, Na^+^ pumping, energy coupling, Mitchell, pyrophosphate

“Scientists frequently debate theories”.

Douglas Allchin

## Introduction

Those of a certain age may remember (and their younger colleagues can read) accounts of the vivid debate in the 1970s surrounding the coupling mechanism involved in oxidative and photo phosphorylation. By that time, Mitchell's chemiosmotic hypothesis had already gained credence, and the debated issue was how a transmembrane H^+^ potential difference drives ATP synthesis by F-type ATP synthases. The major mechanisms that were considered assumed that the membrane (F_o_) and peripheral (F_1_) parts were functionally connected in different ways. Peter Mitchell proposed a “direct coupling” mechanism in which protons are translocated through F_o_ into the catalytic site of F_1_, where they participate directly in ADP phosphorylation and form water as the second product (Mitchell, 1974). Paul Boyer, the proponent of the main competing mechanism, advocated an “indirect coupling” mechanism (successively termed “alternating site”, “binding change”, or “rotational”) that implied that protons transfer their energy to the catalytic site indirectly, *via* distant conformational strain (Boyer, 1997). The debate was resolved in favor of Boyer's mechanism when it became clear that the alternative mechanism is inconsistent with H^+^/ATP stoichiometry and, finally, when the three-dimensional structure of the F-ATPase was determined (Abrahams et al., 1994).

**Figure 1:**
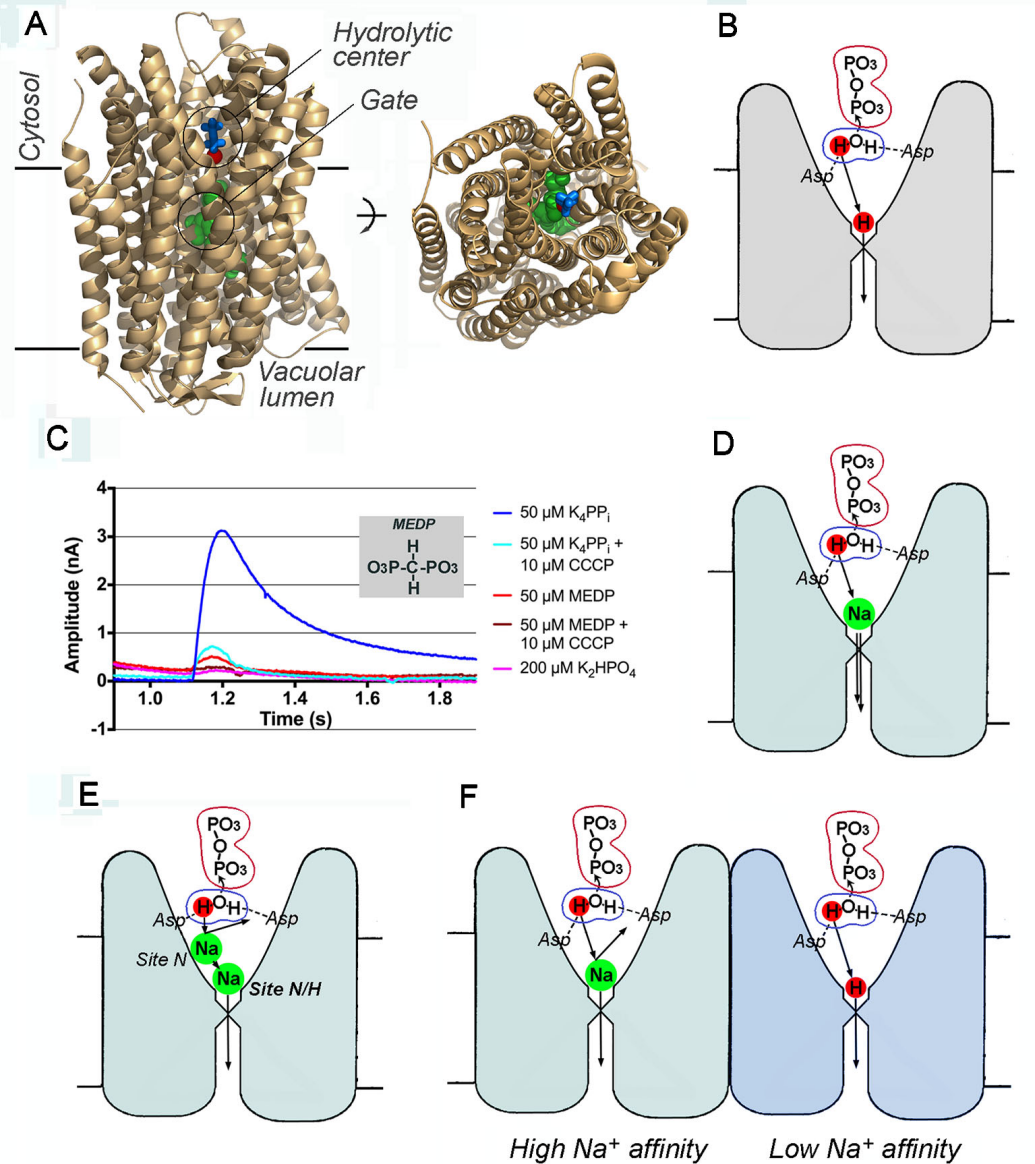
Membrane pyrophosphatase as an H^+^ and Na^+^ transporter. **(A)** Two views of a subunit of *V. radiata* homodimeric H^+^-pyrophosphatase, showing elements of the transport machinery [PDB code: 4A01; Lin et al., 2012)]. The image on the right is a top view from the cytosolic side. Blue sticks, imidodiphosphate; red sphere, water nucleophile (the oxygen atom); green spheres, three gate-forming residues (Arg242, Asp294, and Lys 742); imidodiphosphate-liganded Mg^2+^, and K^+^ ions are not shown. Created with PyMOL (The PyMOL Molecular Graphics System, Version 1.5.0.4, Schrodinger, LLC). **(B)** Mitchell-type coupling of PP_i_ hydrolysis with H^+^ transport in a single subunit. The ions (atoms) directly involved in the transport process are marked by colored circles. Two aspartate residues (Asp287 and Asp731 in *V. radiata* mPPase) coordinate and activate the nucleophilic water molecule during its attack on PP_i_. **(C)** Electrometric traces of *V. radiata* pyrophosphatase-loaded liposomes obtained with a Nanion SURFE^2^R N1 instrument. Currents were recorded following the addition of K_4_PP_i_, methylene diphosphonate (MEDP), and K_2_HPO_4_ in the absence and presence of the protonophore CCCP (carbonyl cyanide *m*-chlorophenyl hydrazone). This panel was reproduced with permission from Shah et al. (2017). **(D)** A billiard-type mechanism of Na^+^ transport at a low Na^+^ concentration. The nucleophile-generated H^+^ pushes out the gate-bound Na^+^ (coordinated by Asp243, Glu246, and Asp703 carboxylates in *T. maritima* mPPase; Li et al., 2016) and passes the gate itself in the same or successive turnover. **(E)** Inhibition of Na^+^ transport by a Na^+^ ion bound at a low-affinity transitory site N. The identities of the residues forming it are yet unknown. **(F)** An alternative mechanism of concurrent Na^+^ and H^+^ transport by different subunits of dimeric Na^+^-PPase. In this mechanism, excess Na^+^ will inhibit H^+^ transport by binding to the pump-loading site of the right subunit, which exhibits a much lower affinity to Na^+^ (strong negative cooperativity).

Now, after several decades, the problem of energy coupling is being revisited in connection with membrane pyrophosphatases (mPPases), ancient transporters that couple H^+^ and Na^+^ transport across biological membranes in plant vacuoles and bacteria to pyrophosphate hydrolysis. mPPases are functional analogs of F-type ATPases and similarly catalyze a direct attack of a water molecule on a phosphorus atom without formation of a phosphorylated intermediate. However, mPPases have a much simpler structure; each of the two identical subunits of mPPase consists of 15−17 transmembrane α-helices, and six of them form the catalytic site on the cytosolic side. H^+^-transporting mPPases (H^+^-PPases) have been known since 1966 (Baltscheffsky et al., 1966; Serrano et al., 2007) and are recognized as contributors to plant stress resistance (Yang et al., 2014). More recent studies have identified an evolutionarily related prokaryotic Na^+^-transporting mPPase lineage (Na^+^-PPases) that can pump both H^+^ and Na^+^ (Malinen et al., 2007; Luoto et al., 2013a; Luoto et al., 2013b). mPPase studies have been further boosted by publication in 2012 of the three-dimensional structures of the H^+^-transporting mPPase from *Vigna radiata* (Lin et al., 2012) (**Figure 1A**) and the Na^+^-transporting mPPase from *Thermotoga maritima* (Kellosalo et al., 2012). Two mechanisms to explain coupling between PP_i_ hydrolysis and H^+^ (Na^+^) pumping, proposed based on these structures, differ principally in the order of hydrolysis and transport events and the role of the proton released by the attacking water nucleophile.

This short treatise on mPPases has three principal purposes. One is to reconsider the available functional data on H^+^-transporting mPPases that favor Mitchell's direct coupling mechanism. The second is to recapitulate modifications to this mechanism to explain Na^+^ transport. And the third is to raise the possibility that mPPases additionally employ elements of Boyer's conformational coupling mechanism.

## Proposed Coupling Mechanisms of H^+^-Transporting mPPase—Pros and Cons

The first coupling mechanism, proposed by Lin et al. (2012) (**Figure 1B**), was essentially an adaptation of Mitchell's hypothesis to mPPases. In the mPPase structure, the presumed water nucleophile is located near the conductance channel, such that the proton released from the attacking water molecule can move to the channel and along it *via* Grotthuss shuttling through a water wire. This proton is thus in the right place at the right time to create high local acidity that drives proton translocation to the other side of the membrane. The mechanism suggested by Lin et al. (2012) therefore assumes that H^+^ transport follows or occurs concurrently with PP_i_ hydrolysis. This mechanism is consistent with the experimentally determined H^+^/PP_i_ coupling ratio of 1 for mPPases (Segami et al., 2018) and, further, predicts that medium H^+^ ions should not compete with the transported H^+^ ion.

An alternative hypothesis (Kellosalo et al., 2012) suggested instead that the transported H^+^ ion passes the gate as a result of PP_i_ binding and that PP_i_ hydrolysis is only required to prepare the transport machinery for the next transport/hydrolysis cycle. This mechanism, named “binding change” (not to be confused with Boyer's “binding change” for F_o_F_1_-ATPase), does not ascribe any specific role to the proton released from the nucleophilic water molecule. Operation of this mechanism in reverse was proposed to explain PP_i_ synthesis by plant mPPases (Regmi et al., 2016).

The proton released by the nucleophilic water is thus the key player in the mechanism of Lin et al., whereas the alternative mechanism ascribes no role to the proton in question, other than being dispersed in the medium. The possibility that this proton is transported in the mechanism of Kellosalo et al. seems unlikely because this would unrealistically presume that the nucleophilic water is converted into a hydroxide ion by means of its coordination to two aspartates. This is reminiscent of the abandoned “charge relay” hypothesis in serine proteases, which assumed similar H^+^ abstraction from a serine hydroxyl (Hedstrom, 2002). Instead, the two aspartates that coordinate the nucleophilic water in mPPases are involved in general acid/base catalysis, as is the case in aspartic proteases (Meek, 1998). Notably, the available structures of several mPPase species formed during the catalytic cycle do not differentiate between these mechanisms, because the reaction intermediates that these structures mimic are common to both mechanisms.

To support the “binding change” hypothesis, Li et al. (2016) and Shah et al. (2017) used a modification of a previously described electrometric assay (Kondrashin et al., 1980) to measure charge movement across the membrane of *V. radiata* mPPase-loaded liposomes in response to non-hydrolyzable PP_i_ analogs (imidodiphosphate and methylene diphosphonate). They indeed observed a small signal of the appropriate sign and interpreted it as an indication that substrate binding alone suffices to transport H^+^ ions across the membrane (**Figure 1C**). However, the authors inexplicably ignored their own observation that PP_i_ produced a 10-times greater signal compared with its analogs (**Figure 1C**), despite similar affinities for mPPase (Baykov et al., 1993). Importantly, the PP_i_ signal arose from a single rather than multiple turnover(s). Indeed, the time required to build up the electrometric signal upon addition of PP_i_ (or its analog) to mPPase-containing liposomes was slightly less than 0.1 s (**Figure 1C**), which is sufficient for only one turnover, based on the turnover number for a purified *V. radiata* mPPase molecule of 11.5 s^-1^ (Segami et al., 2018). In summary, a complete turnover produced a 10-times greater electrometric signal compared to that produced by PP_i_ analog (and seemingly PP_i_) binding. Had the transport event preceded hydrolysis, the signals would have been equal unless the transport stoichiometries for the two ligands differ 10-fold**—**a far-fetched and unlikely scenario. Putting things right side up, the electrometric data strongly support the notion that cation transport is associated with hydrolysis and/or product release, not substrate-binding step in a single turnover.

The low size of the electrometric signals generated by PP_i_ analogs is consistent with charge crossing only part of the membrane thickness (Skulachev et al., 2013), for example, by analog-induced binding of additional Mg^2+^ or H^+^ ions to the active site (the effect of CCCP in **Figure 1C** does not discriminate between primarily transported cations). Alternatively, charged amino acid residues may change their positions in the membrane during the conformational change induced by analog binding (Hsu et al., 2015; Li et al., 2016).

## Billiard-Type Hypothesis of Na^+^ Transport

Although Na^+^-PPases are not found in plants, their study may provide important insights into plant H^+^-PPases because Na^+^-PPases are structurally very similar to H^+^-PPases and can pump both H^+^ and Na^+^ at low (<5 mM) Na^+^ concentrations. The major difference of Na^+^-PPase is the presence of a glutamate residue in the gate that forms a Na^+^-binding site (Kellosalo et al., 2012).

Because Na^+^, unlike the transported H^+^, is not a reaction product and comes from the medium, Na^+^ pumping should employ a different mechanism. The billiard-type hypothesis (Baykov et al., 2013), a logical extension of the mechanism of Lin et al. (2012), posits that the proton released by the nucleophilic water is the major driving force for Na^+^ transport (**Figure 1D**). This proton is assumed to push a bound Na^+^ ion into the ion conductance channel and, at low Na^+^ concentrations, enter the channel itself in place of Na^+^. Notably, neither this nor any other mPPase mechanism found in literature assumes a “one-jump” transfer of cation through the membrane. The particular H^+^ or Na^+^ ion that enters the conductance channel in each turnover exits the channel after *n* turnovers, where *n* is the number of cation-binding sites the cation occupies on its way along the channel. However, a consideration of the pathways through which the cations pass the conductance channel and ionic gate and the associated conformational changes are outside the scope of this article.

The interplay between H^+^ and Na^+^ on their way to the ionic gate appears to involve two cation-binding sites (“N/H” and “N”) in Na^+^-PPases, as indicated by the Na^+^ dependencies of the H^+^- and Na^+^-transporting activities and the effects of substitutions in gate residues (Luoto et al., 2013b). According to these analyses, the pump loading site N/H is associated with the gate and can bind both Na^+^ and H^+^. Its binding constant for Na^+^ lies in the sub-millimolar range, and its occupancy by Na^+^ is required for enzymatic activity. The crystal structure of *T. maritima* Na^+^-PPase (Li et al., 2016) did reveal a gate-bound Na^+^ ion. The other, site N, binds Na^+^ in the millimolar range and presumably acts as a transitory Na^+^-binding site and a filter for H^+^ in the channel (**Figure 1E**). The Na^+^ ion that occupies site N at high Na^+^ concentrations physically or electrostatically disallows H^+^ passage, explaining why dual Na^+^ and H^+^ specificity is observed with most Na^+^-PPases only at low Na^+^ levels (Luoto et al., 2013b). A similar explanation assuming two Na^+^-binding sites was proposed by Holmes et al. (2019).

An alternative possibility is that Na^+^ and H^+^ transport are carried out by different subunits of dimeric Na^+^-PPase binding Na^+^ at a single site per subunit in a negatively cooperative manner because of dimer asymmetry (Artukka et al., 2018; Vidilaseris et al., 2019) (**Figure 1F**). In this mechanism, Na^+^ could inhibit H^+^ transport by occupying both pump-loading sites, resembling the effect of high substrate concentration on enzymatic activity (Artukka et al., 2018).

The “pumping-before-hydrolysis” mechanism of Kellosalo et al. (2012) does not differentiate between H^+^ and Na^+^ and suggests a similar pumping mechanism for both. If, as we saw above, the electrometric data rule out the hypothesis that the transport event precedes substrate hydrolysis in the case of H^+^ pumping, this mechanism is similarly unlikely to operate in Na^+^ pumping. This conclusion is supported by the presence of gate-bound Na^+^ in the complex of TmPPase with imidodiphosphate (Li et al., 2016), but not in the complex with P_i_ (Kellosalo et al., 2012). Similar electrometric measurements with Na^+^-PPases would aid in testing this aspect of the billiard-type mechanism.

## Conclusions and Perspectives

The available data thus indicate that H^+^-PPases operate *via* Mitchell's direct coupling mechanism. But this is only the first milestone in this exciting journey. Recent kinetic data (Artukka et al., 2018) suggest that active sites undergo oscillations between active and inactive conformations during catalysis, a phenomenon resembling the anchor mechanism in watches, and reflecting structural data (Vidilaseris et al., 2019) indicating asymmetrical binding of an allosteric inhibitor to two subunits. This may mean that mPPases combine two mechanisms of energy coupling—Mitchell's direct coupling and Boyer's conformational coupling (its “alternating sites” version), which were antagonists in the debate over F_o_F_1_-ATPase—in one protein.

The interplay between H^+^ and Na^+^ transport activities is another unresolved aspect of mPPase functioning, especially in Na^+^, H^+^-PPases, the group of Na^+^-PPases that pump both Na^+^ and H^+^ at physiological Na^+^ concentrations and, apparently, co-transport both cations in each catalytic cycle (Luoto et al., 2013a). This is thermodynamically permitted in membranes that generate low or moderate electrochemical potential gradients, like those in fermentative bacteria.

Paul Boyer called F_o_F_1_-ATPase a “splendid molecular machine” (Boyer, 1997). This characterization is fully applicable to its predecessor, mPPase, which combines a deceptively simple structure with evolutionary diversity and a multifaceted transport mechanism.

## Author Contributions

The author confirms being the sole contributor of this work and has approved it for publication.

## Funding

This work was supported by a grant from the Russian Science Foundation (research project 19-14-00063).

## Conflict of Interest

The author declares that the research was conducted in the absence of any commercial or financial relationships that could be construed as a potential conflict of interest.
